# The serum proteome of VA-ECMO patients changes over time and allows differentiation of survivors and non-survivors: an observational study

**DOI:** 10.1186/s12967-023-04174-8

**Published:** 2023-05-12

**Authors:** Patrick Malcolm Siegel, Bálint András Barta, Lukas Orlean, Ines Derya Steenbuck, Miguel Cosenza-Contreras, Tobias Wengenmayer, Georg Trummer, Dennis Wolf, Dirk Westermann, Oliver Schilling, Philipp Diehl

**Affiliations:** 1 Department of Cardiology and Angiology, Medical Center - University of Freiburg, Faculty of Medicine, University of Freiburg, Freiburg, Germany; 2 Institute for Surgical Pathology, Medical Center, Faculty of Medicine, University of Freiburg, Freiburg, Germany; 3 Interdisciplinary Medical Intensive Care (IMIT), Medical Center, University of Freiburg, Freiburg, Germany; 4 Department of Cardiovascular Surgery, Medical Center, University of Freiburg, Freiburg, Germany

**Keywords:** ECMO, Proteomics, Mortality, Inflammation, Coagulation, Complement

## Abstract

**Background:**

Veno-arterial extracorporeal membrane oxygenation (VA-ECMO) is applied in patients with refractory hemodynamic failure. Exposure of blood components to high shear stress and the large extracorporeal surfaces in the ECMO circuit trigger a complex inflammatory response syndrome and coagulopathy which are believed to worsen the already poor prognosis of these patients. Mass spectrometry-based proteomics allow a detailed characterization of the serum proteome as it provides the identity and concentration of large numbers of individual proteins at the same time. In this study, we aimed to characterize the serum proteome of patients receiving VA-ECMO.

**Methods:**

Serum samples were collected on day 1 and day 3 after initiation of VA-ECMO. Samples underwent immunoaffinity based depletion for the 14 most abundant serum proteins, in-solution digestion and PreOmics clean-up. A spectral library was built with multiple measurements of a master-mix sample using variable mass windows. Individual samples were measured in data independent acquisition (DIA) mode. Raw files were analyzed by DIA-neural network. Unique proteins were log transformed and quantile normalized. Differential expression analysis was conducted with the LIMMA—R package. ROAST was applied to generate gene ontology enrichment analyses.

**Results:**

Fourteen VA-ECMO patients and six healthy controls were recruited. Seven patients survived. Three hundred and fifty-one unique proteins were identified. One hundred and thirty-seven proteins were differentially expressed between VA-ECMO patients and controls. One hundred and forty-five proteins were differentially expressed on day 3 compared to day 1. Many of the differentially expressed proteins were involved in coagulation and the inflammatory response. The serum proteomes of survivors and non-survivors on day 3 differed from each other according to partial least-squares discriminant analysis (PLS-DA) and 48 proteins were differentially expressed. Many of these proteins have also been ascribed to processes in coagulation and inflammation (e.g., Factor IX, Protein-C, Kallikrein, SERPINA10, SEMA4B, Complement C3, Complement Factor D and MASP-1).

**Conclusion:**

The serum proteome of VA-ECMO patients displays major changes compared to controls and changes from day 1 until day 3. Many changes in the serum proteome are related to inflammation and coagulation. Survivors and non-survivors can be differentiated according to their serum proteomes using PLS-DA analysis on day 3. Our results build the basis for future studies using mass-spectrometry based serum proteomics as a tool to identify novel prognostic biomarkers.

*Trial registration*: DRKS00011106.

**Supplementary Information:**

The online version contains supplementary material available at 10.1186/s12967-023-04174-8.

## Introduction

Veno-arterial extracorporeal membrane oxygenation (VA-ECMO) is applied in patients with hemodynamic failure unresponsive to conventional forms of treatment [1, 2]. Exposure of blood components to high shear stress and the large extracorporeal surface in the ECMO circuit triggers a complex ECMO-associated inflammatory response syndrome and an ECMO-induced coagulopathy which are believed to further aggravate the already poor prognosis of these patients [3]. Important blood components affected include leukocytes [4, 5], platelets [6, 7], the coagulation and the complement system [8].

Mass-spectrometry based proteomics allows a detailed characterization of the serum proteome as it provides the identity and concentration of large numbers of individual proteins at the same time [9]. Recent publications investigating patients with cardiogenic shock, COVID-19, after cardiopulmonary resuscitation, or in an animal model of cardiopulmonary resuscitation and ECMO have demonstrated that the method is highly useful to characterize changes in the serum proteome over time, to assess pathophysiological relationships between proteins and to identify potential prognostic biomarkers [10–13].

In this exploratory study we aimed to characterize the serum proteome of patients receiving VA-ECMO over time, determine differences in the serum proteome between VA-ECMO patients and controls and identify changes in the serum proteome of VA-ECMO patients related to outcome.

## Methods

### Patient recruitment and blood sampling

Patients receiving VA-ECMO on the medical and heart surgical intensive care units of the University Hospital in Freiburg, Germany, were recruited for this study between January and November 2020. Exclusion criteria were age < 18 years, hematological malignancies and hemoglobin < 8 g/dl (to avoid worsening anemia due to blood sampling as required by the Ethics Commission). Clinical and laboratory parameters from ECMO patients were gathered from the electronic patient data management system. Major bleeding was defined as previously described [14]. In surgical patients, bleeding events that were directly related to surgery, were excluded. Survival was defined as discharge from the intensive care unit.

**Fig. 1:**
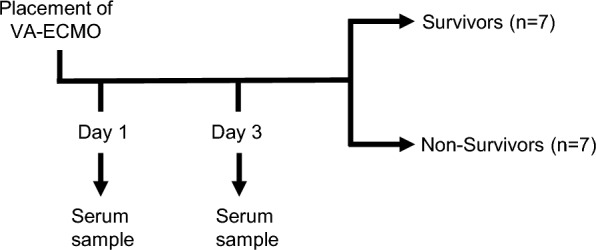
Overview of the study design

Blood was drawn carefully via an arterial catheter into 9 ml serum/gel tubes (S-Monovette^®^ Serum-Gel, Sarstedt, Germany). Blood was sampled on day 1 and day 3 (Fig. 1) of VA-ECMO therapy (i.e.: 6–24 h and 72 ± 12 h after VA-ECMO initiation). Only patients from which blood could be acquired on both days were included in the study. Tubes were kept in an upright position and transported to the laboratory where they were centrifuged as recommended by the manufacturer (2000 g, 10 min, 20 °C). Afterwards, serum aliquots were prepared and stored at − 80 °C until further use.

Blood was also taken from 6 healthy adult control subjects from the antecubital vein. All healthy adult controls were between 20 and 30 years of age, had no history of disease and had not taken any medication in the last 14 days.

### Management of VA-ECMO patients

Indication for VA-ECMO was at the discretion of an experienced ECMO-physician and decided at bedside. Cannulation for VA-ECMO was carried out as previously described [15]. In brief, bifemoral cannulation by Seldinger’s technique without primary surgical cutdown was used. Venous cannulas had a diameter 21 or 23 F and arterial cannulas had a diameter of 15 or 17 F. The following ECMO systems were in use: Maquet Cardiohelp System with an HLS Set Advanced (Maquet Cardiopulmonary GmbH, Rastatt, Germany) and the Stöckert^®^ centrifugal pump (LivaNova PLC, London, United Kingdom). After VA-ECMO placement, patients were managed following standard operating procedures with modifications made at the discretion of an experienced intensivist. Patients without signs of thrombosis or bleeding received unfractionated heparin aiming for an activated partial thromboplastin time of 50–60 s. Minimal targets for hemoglobin and platelet count were 8 g/dl and 100,000/µl.

### Mass spectrometry and bioinformatical analysis

30 µl of thawed supernatant was diluted in buffer (MARS reagent kit buffer A, Agilent) to a total volume of 100 µl and filtered using 0.22 µm filters centrifuged at 16,000 g for 1 min RT. To ensure enhanced reproducibility, high performance liquid chromatography (Äkta explorer, GE Healthcare) was applied for the purpose of protein depletion. Multi affinity removal column (MARS Human-14, Agilent) was employed to remove the 14 most abundant serum proteins (albumin, IgG, IgA, transferrin, haptoglobin, antitrypsin, fibrinogen, alpha_2_-macroglobulin, alpha1-acid glycoprotein, IgM, apolipoproteins AI and AII, C3, and transthyretin) by immunoadsorption from the samples, to allow for identification and quantification of proteins with lower abundance. Unbound proteins were collected, denatured at 90 °C with 1% heat and acid labile surfactant added in 100 mM HEPES buffer. Reductive alkylation was performed using 2-Iodoacetamide 20 mM and Dithiothreitol 2 mM. Proteins were digested into peptides using MS grade trypsin (Promega) added in a 1:50 sample to enzyme ratio at 37 °C overnight. For desalting, PreOmics (Bavaria, Germany) columns were applied. Peptides were eluted with 2% Triethylamine in 80% Acetonitrile (ACN). Buffer was evaporated, peptides were resuspended in 1% formic acid and iRT peptides were added to monitor the performance of liquid chromatography anterior to the mass spectrometer. Measurements of a pooled master sample mixed from all patients and timepoints were used to create spectral library for data-independent acquisition measurements using gas-phase fractionation. All measurements were done on a Q-Exactive Plus (Thermo Scientific, Bremen, Germany). Mass spectra were analyzed using DIANN [16] as described previously [17]. Peptide identification was performed using a human proteome database containing reviewed UniProt sequences without isoforms downloaded from Uniprot on 9th of January 2020. Only proteins that were identified and measured in at least 80% all measured samples were analyzed in this analysis. Residual missing values were imputed with missForest R package [18].

### Statistics

MixOmics [19] R package was employed to execute dimension reduction methods (Principal component analysis (PCA) and Partial Least-Squares Discriminant Analysis (PLS-DA)). Differential expression analysis was performed with LIMMA (R package). ROAST [20] was applied to generate gene ontology enrichment analyses. An adjusted p-value of < 0.05 was considered statistically significant.

## Results

Fourteen patients receiving VA-ECMO were recruited for this study. Five patients were female, 9 patients were male. Seven patients were discharged from the intensive care units and were counted as survivors (Fig. 1 and Table 1). The indication for VA-ECMO was refractory cardiogenic shock: seven patients received VA-ECMO due to protracted cardiogenic shock after cardiovascular surgery. The remaining 7 patients suffered from refractory cardiogenic shock due to medical reasons (e.g., myocardial infarction) and did not receive cardiovascular surgery. Three of these patients received VA-ECMO due to refractory cardiac arrest or post-arrest cardiogenic shock (summarized as extracorporeal cardiopulmonary resuscitation (ECPR) in Table 1). Six patients suffered from major bleeding events, with one event of intracerebral bleeding. However, all major bleeding events were non-lethal. All patients were severely ill as indicated by an elevated SOFA score of 11.0 on day 1.

**Table 1:** Clinical characteristics of VA-ECMO patients on day 1

Parameter	VA-ECMO
Patients, n (%)	14 (100)
Survivors, n (%)	7 (50)
Age, y (IQR)	71 (57–75)
Female, n (%)	5 (36)
Duration of ICU-stay (d, IQR)	12 (7–17)
VA-ECMO Device, n (%)	
Stöckert Sorin	9 (64)
Maquet	5 (36)
Duration of ECMO (d, IQR)	7 (6–7)
ECMO Blood Flow (l/min, IQR)	4.3 (3.3–4.6)
Indication for VA-ECMO, n (%)	
Cardiogenic shock	
Postoperative	7 (50)
Medical	7 (50)
eCPR	3 (21)
Coronary Heart Disease, n (%)	9 (64)
Atrial fibrillation, n (%)	7 (50)
Diabetes mellitus, n (%)	1 (7)
Hypertension, n (%)	3 (21)
Active Smoker, n (%)	2 (14)
Hypercholesterolemia, n (%)	1 (7)
Cancer, n (%)	0 (0)
Acute renal failure, n (%)	10 (71)
Continuous hemodialysis, n (%)	6 (43)
Major bleeding, n (%)	6 (43)
Intracerebral bleeding, n (%)	1 (7)
Minor bleeding, n (%)	4 (29)
Heparin, n (%)	13 (93)
Dual anti-platelet therapy, n (%)	6 (43)
Steroids, n (%)	1 (7)
Mechanical ventilation, n (%)	14 (100)
SOFA score (IQR)	11.0 (8.0–11.0)
Hb (g/dl, IQR)	8.5 (8.3–8.9)
Platelets (× 10^3^/µl, IQR)	109 (79–154)
Creatinine (mg/dl, IQR)	1.3 (1.0–2.5)
Urea (mg/dl, IQR)	51.0 (38.0–71.0)
Bilirubin (mg/dl, IQR)	2.2 (1.6–2.9)
AST (U/l, IQR)	157.0 (58.8–509.3)
ALT (U/l, IQR)	55.0 (21.8–123.8)
Lactate (mmol/l, IQR)	3.6 (1.5–5.3)
Leukocytes (× 10^3^/µl, IQR)	8.9 (6.9–11.2)
CRP (mg/l, IQR)	54.1 (24.5–90.6)
IL-6 (pg/ml, IQR)	370.5 (308.5–784.8)
p_a_O_2_ (mmHg, IQR)	111.0 (81.8–239.0)
p_a_CO_2_ (mmHg, IQR)	38.4 (35.3–50.0)
F_i_O (%, IQR)	47.5 (40.0–50.0)
PEEP (mbar, IQR)	8.0 (7.0–10.0)
Respiratory rate (/min, IQR)	14.0 (11.0–18.0)

Data are presented as median (interquartile range, Q1-Q3) or number of patients (%). Denominator of the percentage is the total number of subjects in the group. Parameters that were closest to the time point of blood sampling for proteome analysis are presented. Major and minor bleeding events are presented as defined by the International Society on Thrombosis and Haemostasis [14]. Macroscopic signs of thrombosis in the extracorporeal circuit were judged as such by an experienced ECMO-physician or ECMO-nurse

ALT; alanine aminotransferase, AST; aspartate aminotransferase, CRP; C-reactive protein, eCPR; extracorporeal cardiopulmonary resuscitation, F_i_O; fraction of inspired oxygen, ICU; intensive care unit, PEEP; positive end-expiratory pressure, SOFA; sequential organ failure assessment score

**Fig. 2:**
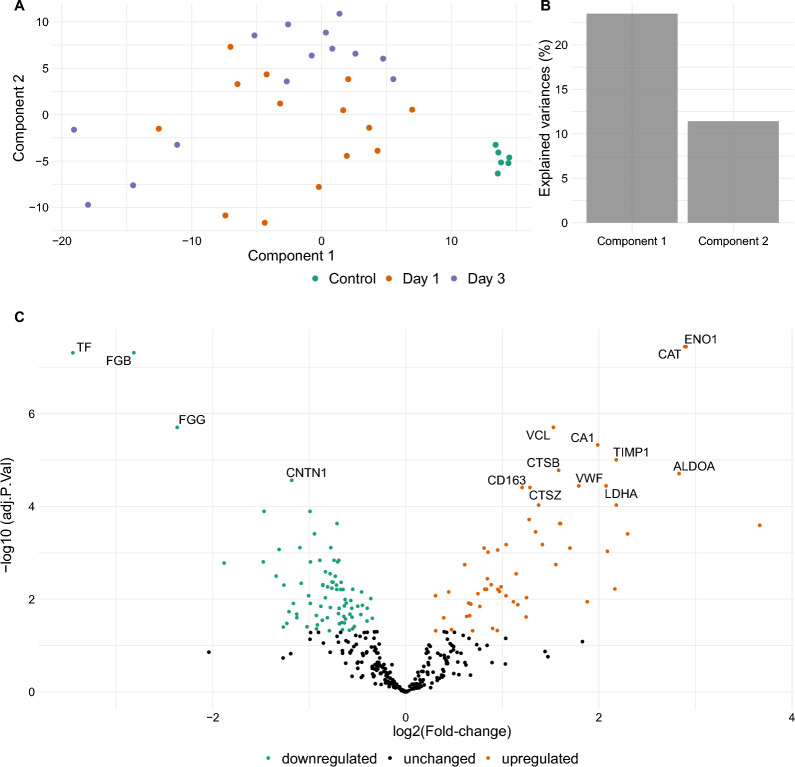
Analysis of the serum proteomes of VA-ECMO patients on day 1 vs. controls. **A** Principal component analysis. **B** Percent of explained variances of components 1 and 2 in the principal component analysis. **C** Volcano plot illustrating differential protein expression in VA-ECMO patients on day 1 vs. controls. Examples of strongly up- and downregulated proteins are given

In total, we identified 351 unique proteins in our samples. Principal component analysis revealed a high similarity of the serum proteomes in the control group which separated clearly from those of VA-ECMO patients (Fig. 2A). Principal components 1 and 2 explained 23.5% and 11.4%, respectively, of the variation in the serum proteomic dataset (Fig. 2B).

**Fig. 3:**
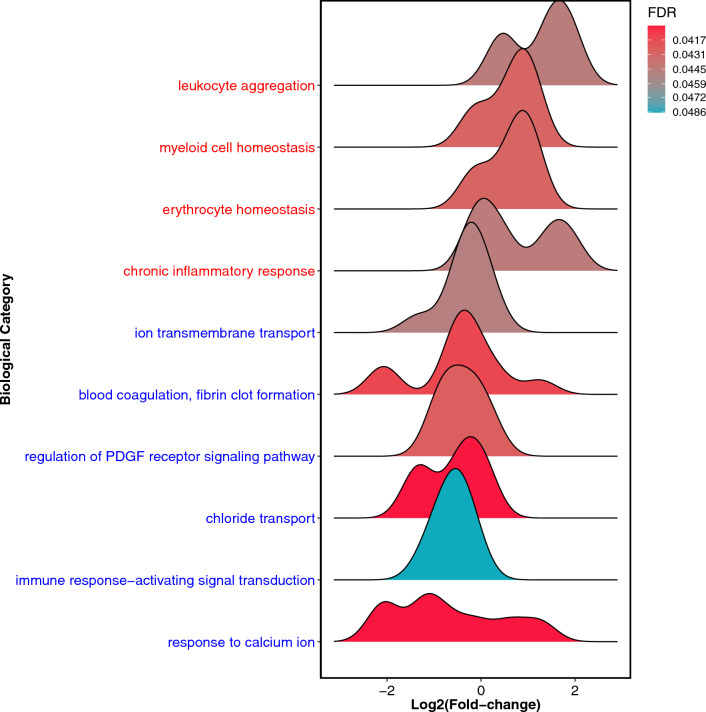
Ridgeline plot illustrating significantly up- (red) or downregulated (blue) GO terms in the serum proteome of VA-ECMO patients on day 1 vs. controls based on Rotation Gene Set Tests (ROAST). GO terms were filtered for those relevant in blood/serum

We then focused our analysis on the comparison of the serum proteomes of VA-ECMO patients on day 1 and controls. Between these two groups, 137 proteins were differentially expressed (Fig. 1C and Additional file 1: Table S1). Many of these upregulated proteins on day 1 in the VA-ECMO group were linked with the inflammatory response explaining enriched gene ontology (GO) terms such as ‘chronic inflammatory response’ and ‘leukocyte aggregation’ (Fig. 3). Several proteins were also downregulated on day 1 compared to controls. Therefore, with respect to the general topic of inflammation, we found that the GO term ‘immune response—activating signal transduction’ was downregulated. Moreover, we also found that proteins linked to the GO term ‘blood coagulation, fibrin clot formation’ were downregulated, e.g., F13A1 ↓, KLKB1 ↓, FBLN1 ↓ and F2 ↓.

**Fig. 4:**
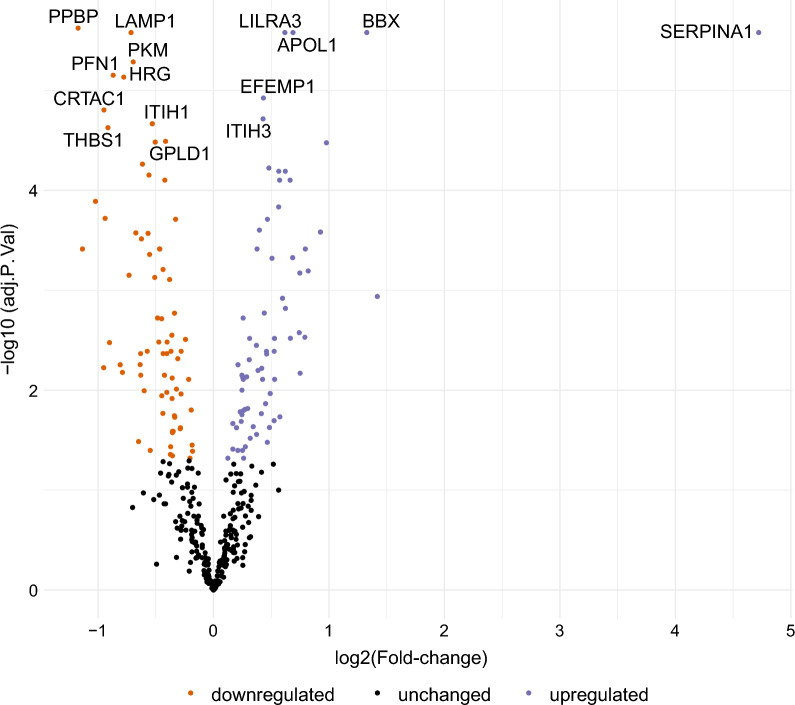
Volcano plot illustrating differential protein expression in VA-ECMO patients on day 3 vs. day 1. Examples of strongly up- and downregulated proteins are given

**Fig. 5:**
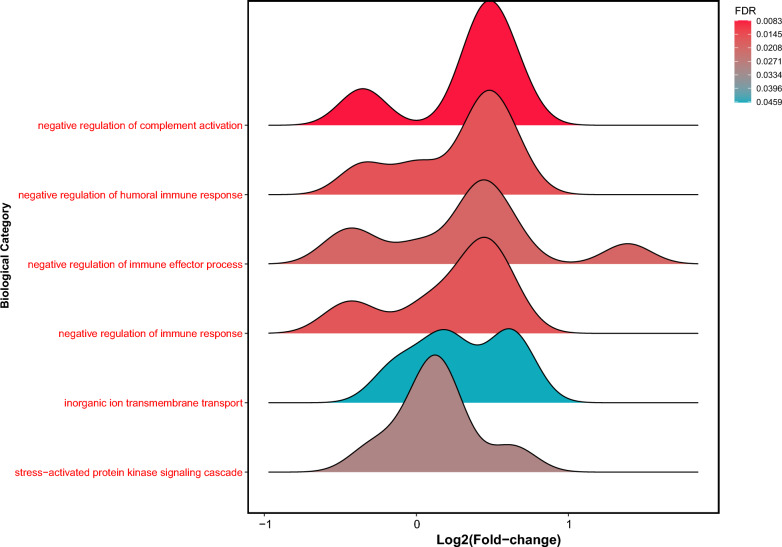
Ridgeline plot illustrating significantly up- (red) or downregulated (blue) GO terms in the serum proteome of VA-ECMO patients on day 3 vs. day 1 based on Rotation Gene Set Tests (ROAST)**.** GO terms were filtered for those relevant in blood/serum

We then assessed the differential protein expression in the serum proteomes of VA-ECMO patients on day 3 vs. day 1 (Fig. 4). In total, 145 proteins were differentially expressed between both groups (Additional file 2: Table S2). One protein in particular, SERPINA1, was highly upregulated on day 3 vs. day 1 (log_2_ fold change 4.7, p < 0.001). Enrichment analysis showed that many proteins linked to downregulation of the immune response were upregulated on day 3 (Fig. 5). GO terms that were upregulated included, for example, ‘negative regulation of complement activation’, ‘negative regulation of humoral immune response’, ‘negative regulation of immune effector process’. Proteins associated with inflammatory processes which were differentially regulated on day 3 vs. day 1 included ICAM-1 & 2 ↑, SERPINA1 ↑, LILRA3 ↑, CRP ↑, CFD ↑, CFI ↑, complement C1S ↑, C2 ↑, C4A ↑, C6 ↑, C8 ↑, C9 ↑. Several proteins with important functions in coagulation (e.g., HRG ↓, THBS1 ↓, PLG ↓, FGA ↑, VWF ↓, F5 ↑, F12 ↓) were also differentially regulated.

**Fig. 6:**
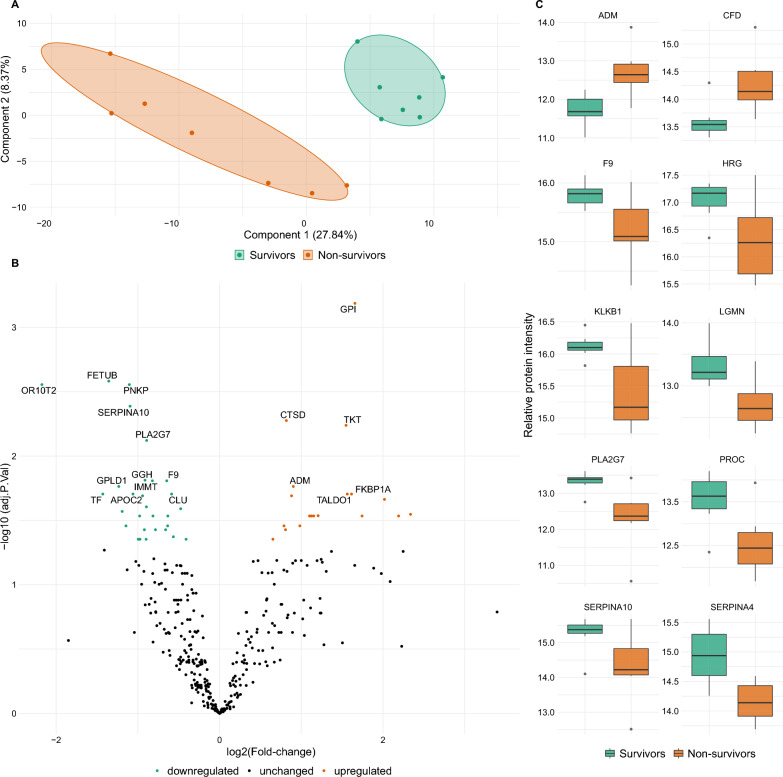
Analysis of the serum proteomes on day 3 of survivors and non-survivors. **A** With the help of PLS-DA the groups of survivors and non-survivors can be differentiated on the graph visually (orange vs. green group). Individual patients are presented as dots in dark green or dark orange within the group ‘areas’ (light green and orange). Therefore, individual patients from the survivor (green) or non-survivor (orange) group are located in different areas on the PLS-DA plot. **B** Volcano plot illustrating differential protein expression in VA-ECMO patients on day 3, non-survivors vs. survivors. **C** Box plots of strongly regulated proteins in non-survivors vs. survivors on day 3 (p < 0.05)

Next, we analyzed differential protein expression in the serum proteomes of non-survivors and survivors. On day 1, only 5 proteins were differentially expressed between both groups (Additional file 3: Table S3). For day 3, PLS-DA indicated that the proteomes of survivors and non-survivors on day 3 are distinguishable (Fig. 6A). Components 1 and 2 accounted for 27.8% and 8.4% of the variances in the proteomic dataset respectively. On day 3, 48 proteins were differentially expressed between non-survivors and survivors (Fig. 6B, Additional file 4: Table S4 and Additional file 5: Table S5). For the non-survivor group on day 3, we noticed decreased abundance of proteins involved in coagulation including F9, KLKB1, PROC, HRG and SERPINA10. Proteins involved in inflammatory processes were also differentially expressed (non-survivors vs. survivors) including C3 ↓, CFD ↑, MASP1 ↓ and PLA2G7 ↓ (Fig. 6C).

## Discussion

This study demonstrates a significantly altered proteome in VA-ECMO patients, which changed during VA-ECMO therapy. Moreover, the serum proteomes of survivors and non-survivors differed substantially on day 3 according to PLS-DA analysis. To the best of our knowledge, this is the first study to perform mass-spectrometry based serum proteomics in a group of adult VA-ECMO patients.

Although other groups have performed mass-spectrometry based serum or plasma proteomics in patients with cardiopulmonary bypass [21–23], results are not directly transferable to VA-ECMO patients as these systems and the patients differ in several aspects, e.g., the shorter duration of cardiopulmonary bypass, the differences in cannulation, the dosage of anticoagulation and the underlying indication (often stable elective patients receiving cardiopulmonary bypass as opposed to critically ill patients with VA-ECMO) [3].

We identified 351 unique proteins, which is similar or higher compared to other recent studies performing serum or plasma proteomics, e.g. Bernhard et al. (n = 388) [10], Distelmaier et al. (n = 299) [12] and Umstead et al. (n = 134) [24]. Our methodology allows for the quantification of a large number of proteins using only a minimal amount of blood (approximately 30 µl) per time point, which is lower than previously described, e.g., by Umstead et al. (~ 100 µl). This is advantageous compared to other blood-based analyses, ELISA or conventional laboratory analyses which usually require comparably large amounts of blood for the limited amount of biological information gathered. Moreover, selecting certain markers for conventional laboratory analysis risks ‘selection bias’, i.e., missing unexpected changes in certain protein levels associated with clinical conditions, the risk of which is avoided or at least minimized by mass-spectrometry based serum proteomics since this method allows characterization and quantification of large numbers of proteins in parallel [25]. Additionally, proteomics is inherently specific as it measures the mass and fragmentation spectra of peptides derived from sequence specific digestion of peptides [9]. This is an advantage, for example, compared to immunoassays, which may suffer from non-specific binding [26].

We demonstrated that the serum proteome of VA-ECMO patients on day 1 differed strongly from the control group, as expected. We therefore did not analyze differences on day 3 of VA-ECMO vs. controls to avoid redundancy. As the initiation of ECMO triggers a strong systemic inflammatory response syndrome, we observed upregulation of the GO term ‘leukocyte aggregation’ associated with inflammation in VA-ECMO patients on day 1 compared to controls. Leukocyte aggregation occurs when activated neutrophils migrate to areas of local inflammation [27]. In this line of evidence, previous studies have demonstrated neutrophil activation in ECMO patients [28, 29]. Neutrophil activation may also be an explanation for the most likely, compensatory upregulation of SERPINA1 in VA-ECMO patients on day 3 vs. day 1, as SERPINA1 has been shown to inhibit the excess of free elastase and neutralize proteinase-3 and myeloperoxidase from neutrophils [30] and is upregulated during the acute phase response [31].

Continuous ECMO is associated with ‘immunoparalysis’ [32], a complex phenomenon which may be the result of multiple factors, such as high shear stress in the extracorporeal circuit, large extracorporeal surfaces and the severe underlying disease and likely increases patient vulnerability towards infection. Immunoparalysis has also been described in patients with cardiopulmonary bypass [33] and sepsis [34]. Leukocyte dysfunction, affecting particularly monocytes and lymphocytes seems to be an important characteristic [4, 5]. In line with these reports, we found that the GO term ‘immune response—activating signal transduction’ associated with a suppressed cellular immune response was downregulated on day 1 vs. controls. This indicates that immune cell dysfunction may be developing as early as day 1 in VA-ECMO patients while there is still an ongoing pro-inflammatory systemic immune response. Whether the humoral immune system, especially the complement system is also dysfunctional is less clear. In our study, enrichment analysis on day 3 revealed GO terms indicating a downregulation of the complement and humoral immune response. Our findings are supported by in-vitro data which demonstrate an initial activation of the complement system [35–37] which is likely followed by an anti-inflammatory response on day 3 as demonstrated in our study.

Bleeding is a severe complication of VA-ECMO and indeed, six patients in our study developed major bleeding complications. Many factors contribute to the ECMO-induced coagulopathy, e.g., thrombocytopenia, anticoagulation [38], platelet dysfunction [6, 7], an acquired von Willebrand Syndrome [39] but also consumption of coagulation factors [40]. Adsorption of coagulation factors and thrombus formation in the extracorporeal circuit, particularly the oxygenators, is one of the main contributors to the consumption of coagulation factors [37, 41]. In our study, this was also reflected on the level of the proteome and translated to the downregulated GO term ‘blood coagulation, fibrin clot formation’ in VA-ECMO patients on day 1 compared to controls.

Overall, our findings are in line with a recent study published by Bernhard et al. who analyzed the serum proteome of pigs before and during VA-ECMO therapy in an experimental resuscitation model. The authors also observed serum proteomic changes related to coagulation and inflammation [10].

Moreover, we report differences in the proteomes of survivors and non-survivors on day 3 as illustrated by PLS-DA analysis. Differential expression analysis indicated several strongly regulated proteins, e.g., GPI was strongly upregulated in non-survivors. GPI’s main function is in glycolysis, but it can also act as a lymphokine [42]. FETUB, on the other hand, is an example of a strongly upregulated protein in survivors. It usually acts as a cysteine protease inhibitor, and has been associated with coronary artery disease [43], a common underlying disease in our cohort. Interestingly, several proteins differentially regulated between survivors and non-survivors are also involved in inflammatory (PLA2G7, MASP1, SEMA4B, C3, CFD) and coagulatory (SERPINA10, F9, KLKB1, HRG, PROC) processes. As coagulation and inflammation are closely linked processes and related to outcome [8], these proteins may serve as future biomarker candidates in validation studies. These results highlight the potential of mass-spectrometry based serum proteomics from a clinical perspective. Since large numbers of proteins are detected and quantified in parallel, the method can help identify previously unknown proteins associated with patient outcome, but it also allows for the identification specific protein patterns in the serum. The identification of such patterns may be further aided by machine learning algorithms and could outperform individual biomarkers [9, 44]. Future studies will have to determine whether the proteins differentially expressed between survivors and non-survivors on day 3 can act as individual biomarkers or may be combined as ‘protein patterns’ to predict survival of VA-ECMO patients. In this line of evidence, researchers recently used quantitative proteomics to perform risk stratification for mortality in patients with cardiogenic shock [45]. Protein patterns identified by mass-spectrometry based serum proteomics have also been used for the early and non-invasive detection of subclinical disease, for example in patients with esophageal carcinoma [46] or patients with carotid atherosclerosis [47]. Protein patterns may also allow to predict the response to certain treatments, which could be a valuable tool for VA-ECMO patients. This is supported by a recent clinical study which demonstrated that mass-spectrometry based serum proteomics could be used to predict the response to immunotherapy in patients with melanoma and non-small cell lung cancer [48].

This study is not without its limitations. As this was an exploratory study, the study was limited to 14 patients. Moreover, power calculations were not performed before study initiation, since the required knowledge of levels of biological heterogeneity and fold changes between conditions was not available before study initiation. Furthermore, due to the study’s observational nature, we cannot exclude effects of the underlying disease on the patients’ serum proteomes. Although we demonstrated that there are significant differences in the serum proteomes of survivors and non-survivors on day 3, future studies will have to determine whether this data can be used to predict the outcome of VA-ECMO patients using a predictive model.

## Conclusion

The serum proteome of VA-ECMO patients displays major changes compared to controls and changes from day 1 until day 3. Many changes in the serum proteome are related to inflammation and coagulation. Survivors and non-survivors can be differentiated according to their serum proteomes using PLS-DA analysis on day 3. Our results build the basis for future studies using mass-spectrometry based serum proteomics as a tool to identify novel prognostic biomarkers.

## Supplementary Information

**Additional file 1.** Differentially expressed proteins day 1 vs. controls

**Additional file 2.** Differentially expressed proteins day 3 vs. day 1

**Additional file 3.** Differentially expressed proteins survivors vs. non-survivors  day 1

**Additional file 4.** Differentially expressed proteins non-survivors vs. survivors day 3

**Additional file 5: Table S5.** Differentially expressed proteins between survivors and non-survivors on day 3 of VA-ECMO therapy. Only proteins that showed significantly different regulation are shown. Examples of their function are provided with references.

## Data Availability

The underlying data are available from the authors upon reasonable request.

## Abbreviations

ECPR: Extracorporeal cardiopulmonary resuscitation
GO term: Gene ontology term
PCA: Principal component analysis
PLS-DA: Partial least-squares discriminant analysis
ROAST: Rotation gene set tests
RT: Room temperature
VA-ECMO: Veno-arterial extracorporeal membrane oxygenation
