# Supplementary material for: The serum proteome of VA-ECMO patients changes over time and allows differentiation of survivors and non-survivors: an observational study
Source: J Transl Med. 2023 May 12;21:319. doi: 10.1186/s12967-023-04174-8 (PMC10176307; doi:10.1186/s12967-023-04174-8)
Supplement: Supplementary file 5 — Additional file 5: Table S5. Differentially expressed proteins between survivors and non-survivors on day 3 of VA-ECMO therapy. Only proteins that showed significantly different regulation are shown. Examples of their function are provided with references. [file 12967_2023_4174_MOESM5_ESM.docx]

| **Gene Name** | **Protein name** | **Function (Selection)** | **Non-Survivor** | **PLS-DA** |
| --- | --- | --- | --- | --- |
| SERPINA10 | Serpin family A member 10 | Inhibits activity of the coagulation protease factor Xa in the presence of PROZ & inhibits factor XIa in the absence of cofactors(1) | DOWN |  |
| F9 | Coagulation factor IX | Part of the intrinsic system activated during ECMO(2). Alterations: Deficiency causes Hemophilia B(3) | DOWN |  |
| KLKB1 | Kallikrein B1 | Surface dependent activation of blood coagulation(4, 5), fibrinolysis(6), inflammation(7) in the ECMO circuit(8). Adsorbed to oxygenator membranes in an in vitro ECMO circuit(9). | DOWN |  |
| HRG | Histidine rich glycoprotein | Multiple functions: e.g., anti-fibrinolytic effect(10), binds to heparin(11), may be procoagulatory, potential biomarker for sepsis(12). Antimicrobial activity(13) | DOWN |  |
| PROC | Protein C | Role in coagulation. Degrads factors V and VIII(14). | DOWN |  |
| PLA2G7 | Phospholipase A2 group VII | Degradation of platelet-activating factor to biologically inactive products(15), increased in inflammatory macrophages and foam cells(16) | DOWN |  |
| MASP1 | MBL associated serine protease 1 | Serine protease involved in complement activation and coagulation(17, 18) | DOWN |  |
| SEMA4B | Semaphorin 4B | Immunoregulatory protein(19), negative regulates basophil functions(20) | DOWN |  |
| C3 | Complement C3 | Central role in the activation of complement system(21). Complement system is activated during ECMO (22) | DOWN | C1 |
| CFD | Complement factor D | Serine peptidase. CFD cleaves factor B bound to C3(b) of the alternative pathway(23).of complement activation, also acts as an adipokine(24) | UP |  |
| FETUB | Fetuin-B | Cysteine protease inhibitor, increased levels associated with coronary artery disease(25) | DOWN | C1 |
| GPI | Glucose-6-phosphate isomerase | Glycolytic enzyme, lymphokine, neuroleukin(26) autoantigen in rheumatoid arthritis(27) | UP |  |
| OR10T2 | Olfactory receptor 10T2 | Olfactory receptor(28) | DOWN | C1 |
| PNKP | Bifunctional polynucleotide 3’ phosphatase/kinase | DNA repair(29) | DOWN |  |
| CTSD | Cathepsin D | Peptidase, protein turnover, activation of hormones, growth factors(30) | UP |  |
| TKT | Transketolase | Catalyzes the interketol transfer between ketoses and aldoses as part of the pentose phosphate pathway(31) | UP |  |
| IMMT | Inner membrane mitochondrial protein | Enables RNA binding activity. Involved in cristae formation(32), preferentially transcribed in heart muscle(33) | DOWN |  |
| ADM | Adrenomedullin | Vasodilation, regulation of hormone secretion, promotion of angiogenesis, and antibacterial activity (E.coli, S.aureus) | UP | C1 |
| GPLD1 | Glycosylphosphatidylinositol specific phospholipase D1 | GPI degrading enzyme(34) | DOWN | C1 |
| CLU | Clusterin | Chaperone involved several processes, e.g., in cell death(35), increased levels in patients with left ventricular remodeling after myocardial infarction(36) | DOWN | C1 |
| TF | Transferrin | Iron transport(37), increase over time in critical COVID 19 patients associated with survival(38) | DOWN | C1 |
| TALDO1 | Transaldolase 1 | key enzyme of the nonoxidative pentose phosphate pathway(39), higher levels in brain dead organ donors associated with acute rejection(40) | UP |  |
| COL18A1 | Collagen type XVIII alpha 1 chain | heparan sulphate proteoglycan, maintains basement membrane integrity(41) | UP | C1 |
| APOM | Apolipoprotein M | Lipid transport, present in HDL and LDL(42), reduced levels in COVID-19 patients(43) | DOWN |  |
| LDHA | Lactate dehydrogenase A | Glycolysis(44), increased in many diseases(45) | UP | C1 |
| FKBP1A | FKBP prolyl isomerase 1A | Member of the immunophilin protein family, peptidylprolyl isomerase activity, binds to immunosuppressants FK506 and rapamycin(46). | UP | C1 |
| GGH | Gamma-glutamyl hydrolase | Hydrolysis of folylpoly-gamma-glutamates and antifolylpoly-gamma-glutamates(47) | DOWN |  |
| APOC2 | Apolipoprotein C2 | Component of chylomicrons, VLDL, and HDL, activates lipoprotein lipase, mutations cause familial chylomicronemia(48) | DOWN |  |
| PMFBP1 | Polyamine modulated factor 1 binding protein 1 | Spermatogenesis(49) | DOWN |  |
| ENO1 | Enolase 1 | Glycolysis, cancer(50) | UP |  |
| TXN | Thioredoxin | Redox reactions(51) | UP |  |
| FSTL1 | Follistatin like 1 | Development(52), Cardioprotective: attenuates hypertrophy following pressure overload(53), prevention of ischemia reperfusion injury in pig (54), loss of epicardial FSTL1 is a maladaptive response to injury (55) | UP |  |
| MCL1 | MCL1 apoptosis regulator, BCL2 family member | Anti-apoptotic(56) | DOWN | C1 |
| IGFBP2 | Insulin like growth factor binding protein 2 | Binds IGF-I and IGF II, promotes several key oncogenic processes (57) | UP |  |
| HP | Haptoglobin | Binds free plasma hemoglobin, may become saturated in ECMO-induced hemolysis(58) | UP | C1 C2 |
| CAT | Catalase | Defense against oxidative stress | UP | C1 |
| LGMN | Legumain | Cysteine protease with specificity for hydrolysis of asparaginyl bonds(59). May promote formation of aortic dissection(60) | DOWN | C1 |
| LDHB | Lactate dehydrogenase B | Glycolysis(44), increased in many diseases(45) | UP | C1 |
| SGSM2 | Small G protein signaling modulator 2 | GTPase activator with activity towards RAB32 and RAB33B, which are regulators of membrane trafficking(61) | DOWN |  |
| NUCB1 | Nucleobindin 1 | Ca^2+^ binding protein, calcium homeostasis, increased during ECMO in pigs(62) | UP |  |
| SERPINA4 | Serpin family A member 4 | Serine proteinase inhibitor(63): anti- and pro-apoptotic, antioxidant and pro-oxidant effects (64) | DOWN | C1 |
| DNAJA1 | Heat shock protein family (Hsp40) member A1 | Heat shock protein 70 cochaperones: facilitate protein folding, trafficking, prevention of aggregation, and proteolytic degradation (65) | DOWN |  |
| PVR | PVR cell adhesion molecule (PVR) (CD155) | Poliovirus receptor, mediates attachment to vitronectin(66), humoral immune response(67) | DOWN |  |
| FBN1 | Fibrillin 1 | Structural support in  connective tissue(68), mutations in FBN-1 gene cause Marfan syndrome(69) | UP | C1 |
| TTN | Titin | Giant protein titin is thought to play major roles in the assembly and function of muscle sarcomeres(70), possible biomarker of muscle atrophy and intensive care unit-acquired weakness in critically ill patients(71) | DOWN |  |
| CRTAC1 | Cartilage acidic protein 1 | Glycosylated extracellular matrix protein that is found in the interterritorial matrix of articular deep zone cartilage, decreased in patients with critical COVID-19 disease (72) | DOWN |  |
| APOF | Apolipoprotein F | True physiological function unknown, most likely lipid transport(73) | DOWN |  |
| LMNB1 | Lamin B1 | Component of the nuclear lamina(74), lamin B1 loss can serve as biomarker of senescence(75) | UP |  |

**Additional Table 5:** Differentially regulated proteins between survivors and non-survivors on day 3 of VA-ECMO therapy. Only proteins that showed significantly different regulation are shown. Examples of their function are provided with references.

**References**

1. Girard TJ, Lasky NM, Tuley EA, Broze GJ, Jr. Protein Z, protein Z-dependent protease inhibitor (serpinA10), and the acute-phase response. J Thromb Haemost. 2013;11(2):375-8.

2. Woodruff RS, Sullenger B, Becker RC. The many faces of the contact pathway and their role in thrombosis. J Thromb Thrombolysis. 2011;32(1):9-20.

3. Biggs R, Douglas AS, Macfarlane RG, Dacie JV, Pitney WR, Merskey. Christmas disease: a condition previously mistaken for haemophilia. Br Med J. 1952;2(4799):1378-82.

4. Wan J, Vadaq N, Konings J, Jaeger M, Kumar V, de Laat B, et al. Kallikrein augments the anticoagulant function of the protein C system in thrombin generation. J Thromb Haemost. 2022;20(1):48-57.

5. Beaubien G, Rosinski-Chupin I, Mattei MG, Mbikay M, Chrétien M, Seidah NG. Gene structure and chromosomal localization of plasma kallikrein. Biochemistry. 1991;30(6):1628-35.

6. Mandle RJ, Jr., Kaplan AP. Hageman-factor-dependent fibrinolysis: generation of fibrinolytic activity by the interaction of human activated factor XI and plasminogen. Blood. 1979;54(4):850-62.

7. Bhoola K, Ramsaroop R, Plendl J, Cassim B, Dlamini Z, Naicker S. Kallikrein and kinin receptor expression in inflammation and cancer. Biol Chem. 2001;382(1):77-89.

8. Ekdahl KN, Huang S, Nilsson B, Teramura Y. Complement inhibition in biomaterial- and biosurface-induced thromboinflammation. Semin Immunol. 2016;28(3):268-77.

9. Große-Berkenbusch K, Avci-Adali M, Arnold M, Cahalan L, Cahalan P, Velic A, et al. Profiling of time-dependent human plasma protein adsorption on non-coated and heparin-coated oxygenator membranes. Biomaterials advances. 2022;139:213014.

10. Lijnen HR, Hoylaerts M, Collen D. Isolation and characterization of a human plasma protein with affinity for the lysine binding sites in plasminogen. Role in the regulation of fibrinolysis and identification as histidine-rich glycoprotein. J Biol Chem. 1980;255(21):10214-22.

11. Hajjar DP, Boyd DB, Harpel PC, Nachman RL. Histidine-rich glycoprotein inhibits the antiproliferative effect of heparin on smooth muscle cells. J Exp Med. 1987;165(3):908-13.

12. Kuroda K, Ishii K, Mihara Y, Kawanoue N, Wake H, Mori S, et al. Histidine-rich glycoprotein as a prognostic biomarker for sepsis. Sci Rep. 2021;11(1):10223.

13. Poon IKH, Patel KK, Davis DS, Parish CR, Hulett MD. Histidine-rich glycoprotein: the Swiss Army knife of mammalian plasma. Blood. 2011;117(7):2093-101.

14. Kisiel W. Human plasma protein C: isolation, characterization, and mechanism of activation by alpha-thrombin. J Clin Invest. 1979;64(3):761-9.

15. Tjoelker LW, Wilder C, Eberhardt C, Stafforini DM, Dietsch G, Schimpf B, et al. Anti-inflammatory properties of a platelet-activating factor acetylhydrolase. Nature. 1995;374(6522):549-53.

16. Ferguson Jane F, Hinkle Christine C, Mehta Nehal N, Bagheri R, DerOhannessian Stephanie L, Shah R, et al. Translational Studies of Lipoprotein-Associated Phospholipase A2 in Inflammation and Atherosclerosis. J Am Coll Cardiol. 2012;59(8):764-72.

17. Golomingi M, Kohler J, Jenny L, Hardy ET, Dobó J, Gál P, et al. Complement lectin pathway components MBL and MASP-1 promote haemostasis upon vessel injury in a microvascular bleeding model. Front Immunol. 2022;13:948190.

18. Choudhary K, Patel PK, Are VN, Makde RD, Hajela K. Mannose-binding lectin-associated serine protease-1 cleaves plasminogen and plasma fibronectin: prefers plasminogen over known fibrinogen substrate. Blood Coagul Fibrinolysis. 2021;32(7):504-12.

19. Suzuki K, Kumanogoh A, Kikutani H. Semaphorins and their receptors in immune cell interactions. Nat Immunol. 2008;9(1):17-23.

20. Nakagawa Y, Takamatsu H, Okuno T, Kang S, Nojima S, Kimura T, et al. Identification of Semaphorin 4B as a Negative Regulator of Basophil-Mediated Immune Responses. The Journal of Immunology. 2011;186(5):2881.

21. Delanghe JR, Speeckaert R, Speeckaert MM. Complement C3 and its polymorphism: biological and clinical consequences. Pathology. 2014;46(1):1-10.

22. Plötz FB, van Oeveren W, Bartlett RH, Wildevuur CR. Blood activation during neonatal extracorporeal life support. J Thorac Cardiovasc Surg. 1993;105(5):823-32.

23. Ricklin D, Hajishengallis G, Yang K, Lambris JD. Complement: a key system for immune surveillance and homeostasis. Nat Immunol. 2010;11(9):785-97.

24. White RT, Damm D, Hancock N, Rosen BS, Lowell BB, Usher P, et al. Human adipsin is identical to complement factor D and is expressed at high levels in adipose tissue. J Biol Chem. 1992;267(13):9210-3.

25. Zhu K, Wang Y, Shu P, Zhou Q, Zhu J, Zhou W, et al. Increased serum levels of fetuin B in patients with coronary artery disease. Endocrine. 2017;58(1):97-105.

26. Haga A, Niinaka Y, Raz A. Phosphohexose isomerase/autocrine motility factor/neuroleukin/maturation factor is a multifunctional phosphoprotein. Biochim Biophys Acta. 2000;1480(1-2):235-44.

27. Matsumoto I, Kurata I, Ohyama A, Kawaguchi H, Ebe H, Osada A, et al. Revisit of autoimmunity to glucose-6-phosphate isomerase in experimental and rheumatoid arthritis. Mod Rheumatol. 2020;30(2):232-8.

28. Malnic B, Godfrey PA, Buck LB. The human olfactory receptor gene family. Proc Natl Acad Sci U S A. 2004;101(8):2584-9.

29. Whitehouse CJ, Taylor RM, Thistlethwaite A, Zhang H, Karimi-Busheri F, Lasko DD, et al. XRCC1 stimulates human polynucleotide kinase activity at damaged DNA termini and accelerates DNA single-strand break repair. Cell. 2001;104(1):107-17.

30. Benes P, Vetvicka V, Fusek M. Cathepsin D—Many functions of one aspartic protease. Crit Rev Oncol Hematol. 2008;68(1):12-28.

31. Mitschke L, Parthier C, Schröder-Tittmann K, Coy J, Lüdtke S, Tittmann K. The crystal structure of human transketolase and new insights into its mode of action. J Biol Chem. 2010;285(41):31559-70.

32. von der Malsburg K, Müller Judith M, Bohnert M, Oeljeklaus S, Kwiatkowska P, Becker T, et al. Dual Role of Mitofilin in Mitochondrial Membrane Organization and Protein Biogenesis. Dev Cell. 2011;21(4):694-707.

33. Icho T, Ikeda T, Matsumoto Y, Hanaoka F, Kaji K, Tsuchida N. A novel human gene that is preferentially transcribed in heart muscle. Gene. 1994;144(2):301-6.

34. Hoener MC, Brodbeck U. Phosphatidylinositol-glycan-specific phospholipase D is an amphiphilic glycoprotein that in serum is associated with high-density lipoproteins. Eur J Biochem. 1992;206(3):747-57.

35. Ahuja HS, Tenniswood M, Lockshin R, Zakeri ZF. Expression of clusterin in cell differentiation and cell death. Biochem Cell Biol. 1994;72(11-12):523-30.

36. Turkieh A, Fertin M, Bouvet M, Mulder P, Drobecq H, Lemesle G, et al. Expression and Implication of Clusterin in Left Ventricular Remodeling After Myocardial Infarction. Circ Heart Fail. 2018;11(6):e004838.

37. Gkouvatsos K, Papanikolaou G, Pantopoulos K. Regulation of iron transport and the role of transferrin. Biochimica et Biophysica Acta (BBA) - General Subjects. 2012;1820(3):188-202.

38. Demichev V, Tober-Lau P, Nazarenko T, Lemke O, Kaur Aulakh S, Whitwell HJ, et al. A proteomic survival predictor for COVID-19 patients in intensive care. PLOS Digital Health. 2022;1(1):e0000007.

39. Banki K, Hutter E, Colombo E, Gonchoroff NJ, Perl A. Glutathione Levels and Sensitivity to Apoptosis Are Regulated by Changes in Transaldolase Expression *. J Biol Chem. 1996;271(51):32994-3001.

40. Lukac J, Dhaygude K, Saraswat M, Joenväärä S, Syrjälä SO, Holmström EJ, et al. Plasma proteome of brain-dead organ donors predicts heart transplant outcome. The Journal of Heart and Lung Transplantation. 2022;41(3):311-24.

41. Heljasvaara R, Aikio M, Ruotsalainen H, Pihlajaniemi T. Collagen XVIII in tissue homeostasis and dysregulation — Lessons learned from model organisms and human patients. Matrix Biol. 2017;57-58:55-75.

42. Xu N, Dahlbäck B. A novel human apolipoprotein (apoM). J Biol Chem. 1999;274(44):31286-90.

43. Begue F, Tanaka S, Mouktadi Z, Rondeau P, Veeren B, Diotel N, et al. Altered high-density lipoprotein composition and functions during severe COVID-19. Sci Rep. 2021;11(1):2291.

44. Schumann G, Bonora R, Ceriotti F, Clerc-Renaud P, Ferrero CA, Férard G, et al. IFCC primary reference procedures for the measurement of catalytic activity concentrations of enzymes at 37 degrees C. Part 3. Reference procedure for the measurement of catalytic concentration of lactate dehydrogenase. Clin Chem Lab Med. 2002;40(6):643-8.

45. Drent M, Cobben NA, Henderson RF, Wouters EF, van Dieijen-Visser M. Usefulness of lactate dehydrogenase and its isoenzymes as indicators of lung damage or inflammation. Eur Respir J. 1996;9(8):1736.

46. Siekierka JJ, Wiederrecht G, Greulich H, Boulton D, Hung SH, Cryan J, et al. The cytosolic-binding protein for the immunosuppressant FK-506 is both a ubiquitous and highly conserved peptidyl-prolyl cis-trans isomerase. J Biol Chem. 1990;265(34):21011-5.

47. Yao R, Schneider E, Ryan TJ, Galivan J. Human gamma-glutamyl hydrolase: cloning and characterization of the enzyme expressed in vitro. Proc Natl Acad Sci U S A. 1996;93(19):10134-8.

48. Jong MC, Hofker MH, Havekes LM. Role of ApoCs in Lipoprotein Metabolism. Arterioscler Thromb Vasc Biol. 1999;19(3):472-84.

49. Zhu F, Liu C, Wang F, Yang X, Zhang J, Wu H, et al. Mutations in PMFBP1 Cause Acephalic Spermatozoa Syndrome. Am J Hum Genet. 2018;103(2):188-99.

50. Zhu X, Miao X, Wu Y, Li C, Guo Y, Liu Y, et al. ENO1 promotes tumor proliferation and cell adhesion mediated drug resistance (CAM-DR) in Non-Hodgkin's Lymphomas. Exp Cell Res. 2015;335(2):216-23.

51. Lee S, Kim SM, Lee RT. Thioredoxin and thioredoxin target proteins: from molecular mechanisms to functional significance. Antioxid Redox Signal. 2013;18(10):1165-207.

52. Sylva M, Moorman AF, van den Hoff MJ. Follistatin-like 1 in vertebrate development. Birth defects research Part C, Embryo today : reviews. 2013;99(1):61-9.

53. Shimano M, Ouchi N, Nakamura K, van Wijk B, Ohashi K, Asaumi Y, et al. Cardiac myocyte follistatin-like 1 functions to attenuate hypertrophy following pressure overload. Proc Natl Acad Sci U S A. 2011;108(43):E899-906.

54. Ogura Y, Ouchi N, Ohashi K, Shibata R, Kataoka Y, Kambara T, et al. Therapeutic impact of follistatin-like 1 on myocardial ischemic injury in preclinical models. Circulation. 2012;126(14):1728-38.

55. Wei K, Serpooshan V, Hurtado C, Diez-Cuñado M, Zhao M, Maruyama S, et al. Epicardial FSTL1 reconstitution regenerates the adult mammalian heart. Nature. 2015;525(7570):479-85.

56. Kelly GL, Strasser A. Toward Targeting Antiapoptotic MCL-1 for Cancer Therapy. Annual Review of Cancer Biology. 2020;4(1):299-313.

57. Li T, Forbes ME, Fuller GN, Li J, Yang X, Zhang W. IGFBP2: integrative hub of developmental and oncogenic signaling network. Oncogene. 2020;39(11):2243-57.

58. Sniderman J, Monagle P, Annich GM, MacLaren G. Hematologic concerns in extracorporeal membrane oxygenation. Research and Practice in Thrombosis and Haemostasis. 2020;4(4):455-68.

59. Chen JM, Dando PM, Rawlings ND, Brown MA, Young NE, Stevens RA, et al. Cloning, isolation, and characterization of mammalian legumain, an asparaginyl endopeptidase. J Biol Chem. 1997;272(12):8090-8.

60. Pan L, Bai P, Weng X, Liu J, Chen Y, Chen S, et al. Legumain Is an Endogenous Modulator of Integrin αvβ3 Triggering Vascular Degeneration, Dissection, and Rupture. Circulation. 2022;145(9):659-74.

61. Yang H, Sasaki T, Minoshima S, Shimizu N. Identification of three novel proteins (SGSM1, 2, 3) which modulate small G protein (RAP and RAB)-mediated signaling pathway. Genomics. 2007;90(2):249-60.

62. Bernhard P, Bretthauer BA, Brixius SJ, Bügener H, Groh JE, Scherer C, et al. Serum proteome alterations during conventional and extracorporeal resuscitation in pigs. J Transl Med. 2022;20(1):238.

63. Chai KX, Chen LM, Chao J, Chao L. Kallistatin: a novel human serine proteinase inhibitor. Molecular cloning, tissue distribution, and expression in Escherichia coli. J Biol Chem. 1993;268(32):24498-505.

64. Chao J, Li P, Chao L. Kallistatin: double-edged role in angiogenesis, apoptosis and oxidative stress. Biol Chem. 2017;398(12):1309-17.

65. Qiu XB, Shao YM, Miao S, Wang L. The diversity of the DnaJ/Hsp40 family, the crucial partners for Hsp70 chaperones. Cell Mol Life Sci. 2006;63(22):2560-70.

66. Lange R, Peng X, Wimmer E, Lipp M, Bernhardt G. The poliovirus receptor CD155 mediates cell-to-matrix contacts by specifically binding to vitronectin. Virology. 2001;285(2):218-27.

67. Maier MK, Seth S, Czeloth N, Qiu Q, Ravens I, Kremmer E, et al. The adhesion receptor CD155 determines the magnitude of humoral immune responses against orally ingested antigens. Eur J Immunol. 2007;37(8):2214-25.

68. Maslen CL, Corson GM, Maddox BK, Glanville RW, Sakai LY. Partial sequence of a candidate gene for the Marfan syndrome. Nature. 1991;352(6333):334-7.

69. Ramachandra CJ, Mehta A, Guo KW, Wong P, Tan JL, Shim W. Molecular pathogenesis of Marfan syndrome. Int J Cardiol. 2015;187:585-91.

70. Tskhovrebova L, Trinick J. Roles of titin in the structure and elasticity of the sarcomere. Journal of biomedicine & biotechnology. 2010;2010:612482.

71. Nakanishi N, Tsutsumi R, Hara K, Matsuo M, Sakaue H, Oto J. Urinary Titin N-Fragment as a Biomarker of Muscle Atrophy, Intensive Care Unit-Acquired Weakness, and Possible Application for Post-Intensive Care Syndrome. Journal of clinical medicine. 2021;10(4).

72. Byeon SK, Madugundu AK, Garapati K, Ramarajan MG, Saraswat M, Kumar-M P, et al. Development of a multiomics model for identification of predictive biomarkers for COVID-19 severity: a retrospective cohort study. The Lancet Digital Health. 2022;4(9):e632-e45.

73. Lagor WR, Fields DW, Khetarpal SA, Kumaravel A, Lin W, Weintraub N, et al. The effects of apolipoprotein F deficiency on high density lipoprotein cholesterol metabolism in mice. PLoS One. 2012;7(2):e31616.

74. Lin F, Worman HJ. Structural organization of the human gene (LMNB1) encoding nuclear lamin B1. Genomics. 1995;27(2):230-6.

75. Freund A, Laberge R-M, Demaria M, Campisi J. Lamin B1 loss is a senescence-associated biomarker. Mol Biol Cell. 2012;23(11):2066-75.
